# Early Functional Impairment in Smokers with CT-Detected Emphysema: Spirometry Provides Complementary Physiological Information in Lung Cancer Screening

**DOI:** 10.3390/biomedicines14040847

**Published:** 2026-04-08

**Authors:** Sanja Dimic-Janjic, Ivana Buha, Jelena Cvejic, Nikola Kostadinovic, Slavko Stamenic, Anka Postic, Ana Ratkovic, Kristina Stosic-Markovic, Ivana Sekulovic-Radovanovic, Marija Vukoja, Nikola Trboljevac, Lidija Isovic, Ruza Stevic, Nikola Colic, Katarina Lukic, Spasoje Popevic, Natasa Djurdjevic, Milan Savic, Nikola Subotic, Mihailo Stjepanovic

**Affiliations:** 1 Faculty of Medicine, University of Belgrade, 11000 Belgrade, Serbia; 2 Clinic for Pulmonology, University Clinical Center of Serbia, 11000 Belgrade, Serbia; 3 Faculty of Medicine, University of Novi Sad, 21000 Novi Sad, Serbia; 4 Institute for Pulmonary Diseases of Vojvodina, 21000 Novi Sad, Serbia; 5 Center for Radiology, University Clinical Center of Serbia, 11000 Belgrade, Serbia; 6 Clinic for Thoracic Surgery, University Clinical Center of Serbia, 11000 Belgrade, Serbia

**Keywords:** lung cancer screening, CT-detected emphysema, spirometry, small airways dysfunction, flow–volume curve, airflow limitation

## Abstract

**Background**: Low-dose computed tomography (LDCT) lung cancer screening (LCS) frequently identifies emphysema in high-risk smokers. However, the extent to which CT-detected emphysema reflects underlying physiological impairment remains uncertain. We evaluated whether spirometry can detect functional abnormalities in this population beyond structural imaging findings. **Methods**: This cross-sectional study included 323 individuals with LDCT- detected emphysema and no lung cancer or prior chronic respiratory diseases within a screening cohort (*n* = 3076). Participants underwent pre-bronchodilator spirometry and symptom assessments (COPD Assessment test (CAT) and Modified Medical Research Council (mMRC) Dyspnea Scale). Pre-bronchodilator airflow limitation was defined as forced expiratory volume in one second to forced vital capacity ratio (FEV_1_/FVC) < 0.70. Small airways dysfunction was defined by ≥2 reduced mid-expiratory flow parameters (<60% predicted). Flow–volume curve morphology was assessed qualitatively. **Results**: Pre-bronchodilator airflow limitation was observed in 45.2% of participants, predominantly mild. Small-airway dysfunction was present in 52%, and an abnormal flow–volume curve morphology in 67.5%. Notably, functional abnormalities were frequently observed despite preserved FEV_1_. Symptom burden was low, with only 7.7% of participants reporting clinically significant symptoms. Functional impairments often overlapped and were common in minimally symptomatic individuals. **Conclusions**: In a lung cancer screening (LCS) cohort with CT-detected emphysema, functional abnormalities are frequently observed, including in individuals with preserved FEV_1_ and minimal symptoms. Spirometry provides additional physiological insight beyond structural imaging; however, these findings are descriptive and should not be interpreted as diagnostic of COPD. Further studies are needed to determine their clinical relevance.

## 1. Introduction

Lung cancer screening (LCS) using low-dose computed tomography (LDCT) has become a well-established strategy for reducing lung cancer–related mortality in high-risk smokers [1]. In addition to early cancer detection, LDCT frequently identifies incidental pulmonary abnormalities, most notably emphysema, which is strongly associated with smoking exposure and adverse respiratory outcomes [2]. Increasing evidence suggests that radiological emphysema represents an early manifestation of smoking-related lung injury and might occur before the clinical signs of chronic obstructive pulmonary disease (COPD) become evident [3,4].

COPD is traditionally diagnosed using spirometric criteria based on airflow obstruction, but structural and functional abnormalities often develop long before classical diagnostic thresholds are reached. Small airways dysfunction is increasingly recognized as an important feature of early smoking-related lung disease and may serve as a sensitive marker of early physiological impairment [5].

LCS populations represent a unique opportunity to investigate early smoking-related lung abnormalities, as participants are typically current or former smokers with substantial exposure but often minimal respiratory symptoms. Previous studies have demonstrated a high prevalence of undiagnosed airflow limitation and emphysema in screening cohorts, but the relationship between radiological emphysema, small airways dysfunction, spirometric abnormalities, and symptom burden remains incompletely characterized [6,7,8]. In particular, the extent to which CT-detected emphysema reflects underlying physiological impairment beyond standard spirometric measures is still uncertain.

In this context, we hypothesized that combining spirometry and symptom assessment with LDCT findings may improve the identification of individuals with early physiological abnormalities and increased risk of future COPD within a screening setting. Such an approach may enable recognition of physiologically vulnerable individuals before the onset of clinically significant disease or symptom-driven healthcare contact.

## 2. Materials and Methods

### 2.1. Population and Study Design

This cross-sectional study involved 323 individuals with LDCT-detected emphysema and no lung cancer or other chronic respiratory diseases, identified within a lung cancer screening cohort (LCS) (*n* = 3076). The aim of this study was to determine whether spirometry, combined with LDCT-detected emphysema and respiratory symptom assessment, could identify early physiological abnormalities in this high-risk population. We hypothesized that adding spirometry to LDCT-based screening may provide complementary physiological information beyond imaging alone and may help identify individuals at increased risk of future COPD.

The study was conducted within a screening setting, where methodological choices were guided by feasibility and workflow considerations rather than by a full diagnostic respiratory evaluation.

The study was conducted at the University Clinical Center of Serbia, Clinic for Pulmonology, in Belgrade. During the initial phase, from November 2024 to May 2025, a group of 3076 participants aged 50 and older with a smoking history of more than 30 years underwent LCS using LDCT. In addition to assessing lung cancer, radiological evaluations for emphysema were also performed. In the second phase, from May 2025 to July 2025, all individuals with prior radiological evidence of emphysema and no lung cancer or other imaging abnormalities were contacted by phone to undergo spirometry, and all invited participants completed the assessment. Exclusion criteria for spirometry included any known chronic respiratory illness.

A current smoker was defined as someone who either smokes every day (a daily smoker) or currently smokes but not every day (an occasional or nondaily smoker) [9]. Symptoms were assessed using the Modified Medical Research Council Dyspnea Scale (mMRC) and the COPD Assessment Test (CAT), both translated and validated [10]. Functional domains, including pre-bronchodilator airflow limitation, small airways dysfunction, flow-volume curve morphology, and symptom burden, were evaluated independently. The overlap among these features was explored descriptively and illustrated conceptually, without assuming mutual exclusivity or a hierarchical progression of the disease.

### 2.2. Radiological Assessment of Emphysema

LDCT examinations were performed on a Siemens Definition Edge 128-slice scanner using a low-dose protocol (20 mA, 100 kV). Images were acquired during a single deep inspiratory breath-hold and reconstructed with 1 mm slice thickness using a lung window (BF37 kernel). Acquisition parameters and reconstruction settings were standardized across all participants as part of the lung cancer screening protocol.

Emphysema was defined using a standard densitometric threshold of −950 HU on inspiratory CT scans, which is a widely accepted criterion for identifying emphysematous lung tissue [11,12,13,14].

In accordance with the screening-oriented design of the study, participants were classified dichotomously as having or not having radiological evidence of emphysema, rather than being stratified according to emphysema extent. The aim was not to quantify emphysema burden but to identify individuals with structural lung abnormalities within a screening setting.

Radiological classification was performed using a structured assessment based on standardized criteria. Detailed quantitative analysis of emphysema extent (e.g., low-attenuation area percentage, LAA-950%) and additional imaging, such as expiratory scans, were not performed, as the CT protocol was designed primarily for lung cancer screening and reflects real-world clinical practice.

### 2.3. Pulmonary Function and Symptom Assessment

Pre-bronchodilator spirometry was performed by a trained technician using a laboratory spirometer, Vyntus SPIRO spirometer (Vyaire Medical, Hochberg, Germany), with SentrySuite software V. 3.10, in accordance with ERS and American Thoracic Society (ATS) criteria [15,16,17,18]. Spirometry reference values were based on the Global Lung Initiative (GLI) 2012 equations, using the appropriate reference population. Daily calibration of the spirometer was performed according to manufacturer recommendations using a standardized calibration syringe. Spirometry quality control followed ERS/ATS standards, including at least three acceptable maneuvers with repeatability within 150 mL for FEV_1_ and FVC [16].

Pre-bronchodilator spirometry was performed as a pragmatic first-line functional assessment within a lung cancer screening (LCS) setting to characterize baseline physiological status within a screening population rather than to establish a diagnosis of COPD. Post-bronchodilator testing was not included due to workflow and feasibility constraints inherent to large-scale screening programs, in which rapid evaluation of a large volume of participants is required. Spirometry was used to assess physiological abnormalities within the screening cohort rather than to establish a definitive diagnosis of COPD. Assessed parameters included FEV_1_ (forced expiratory volume in one second), FVC (forced vital capacity), FEV_1_/FVC ratio, peak expiratory flow (PEF), and mid-expiratory flow indices (FEF_25–75_, MEF_25_, MEF_50_, and MEF_75_).

Pre-bronchodilator airflow limitation was defined as FEV_1_/FVC < 0.70. Because post-bronchodilator testing was not performed, this measure was interpreted as physiological airflow limitation rather than confirmed persistent airflow obstruction. FEV_1_ percentage predicted was used to describe the severity of pre-bronchodilator airflow limitation among participants, according to GOLD (Global Initiative for Chronic Obstructive Lung Disease) spirometric grades as GOLD 1 (Mild) (FEV_1_ ≥ 80%), GOLD 2 (Moderate) (50% ≤ FEV_1_ < 80%), GOLD 3 (Severe) (30% ≤ FEV_1_ < 50%), and GOLD 4 (Very Severe) (FEV_1_ < 30% predicted, or <50% with respiratory failure) [19].

Small airways dysfunction was defined as the presence of at least two of the following three parameters below 60% of predicted: FEF_25–75_, MEF_50_, and MEF_75_. This threshold was chosen to improve specificity and reduce the influence of physiological variability of isolated mid-expiratory flow indices, in line with previously used functional criteria [15,20,21,22,23,24].

Flow–volume curve morphology was assessed qualitatively using visual inspection of maximal expiratory and inspiratory maneuvers. A normal expiratory curve was defined as a rapid rise to peak expiratory flow followed by a smooth descending limb without marked concavity. Curves were classified as pathological in the presence of characteristic abnormalities, including expiratory concavity (“scooping”), irregular oscillations (“saw-tooth” pattern), reduced peak expiratory flow, or other deviations from the expected contour of a normal curve [15,16,25,26]. Curves were displayed according to ATS recommendations, and the software allowed comparison with the predicted reference curve [27].

The assessment was performed independently by an experienced physician trained in pulmonary function testing. Formal interobserver variability analysis was not performed, which is acknowledged as a limitation.

### 2.4. Statistical Analysis

Categorical variables were presented as absolute counts and percentages. Continuous variables were summarized using either the mean ± standard deviation or the median (min–max), depending on distribution and variable characteristics. Parametric data are presented as mean ± SD, while non-normally distributed variables and mid-expiratory flow parameters are presented as median values. Normality was assessed using both statistical (Shapiro–Wilk test) and graphical (histogram and box plot) methods. Statistical significance was set at *p* < 0.05. The analysis was conducted using IBM SPSS version 29.

## 3. Results

### 3.1. Study Population

During the study period, 3076 individuals underwent lung cancer screening. Among them, 323 patients (10.5%) had radiological evidence of emphysema on LDCT and were included in the analysis. The mean age was 65.2 ± 7.6 years, with equal representation of males and females (50.5% vs. 49.5%), and a mean BMI of 26.2 ± 4.4 kg/m^2^. Pre-bronchodilator spirometry characteristics are presented in Table 1.

### 3.2. Spirometry Characteristics

Participants had preserved mean FVC (114.5 ± 17.2%) and FEV_1_ (98.9 ± 18.7%), while the mean FEV_1_/FVC ratio was reduced (69.6 ± 8.5). Pre-bronchodilator airflow limitation (FEV_1_/FVC < 0.70) was present in 45.2% (*n* = 146). Indicators of small airways dysfunction were also frequent: 52.0% had FEF_25–75_ < 60%, and 67.5% (*n* = 218) exhibited pathological flow–volume curve morphology, including many individuals with preserved FEV_1_.

#### Severity of Airflow Limitation

**Figure 1:**
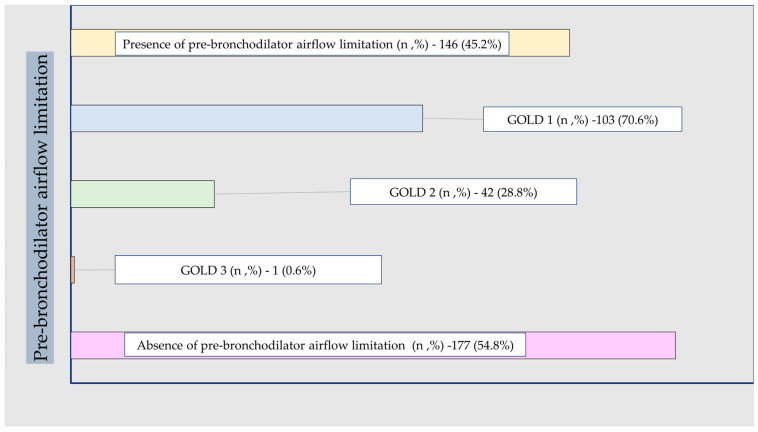
Distribution of pre-bronchodilator airflow limitation severity (GOLD stages).

The prevalence and severity of pre-bronchodilator airflow limitation, according to GOLD spirometric stages 1–4, are shown in Figure 1. Among participants with pre-bronchodilator airflow limitation, 70.6% were classified as GOLD 1, 28.8% as GOLD 2, and 0.6% as GOLD 3. Thus, pre-bronchodilator airflow limitation was predominantly mild. In parallel, 52% of participants had reduced FEF_25–75,_ and 67.5% had pathological flow–volume curves, indicating that physiological abnormalities extended beyond the FEV_1_/FVC criterion alone.

### 3.3. Symptoms (MMRC and CAT)

**Figure 2:**
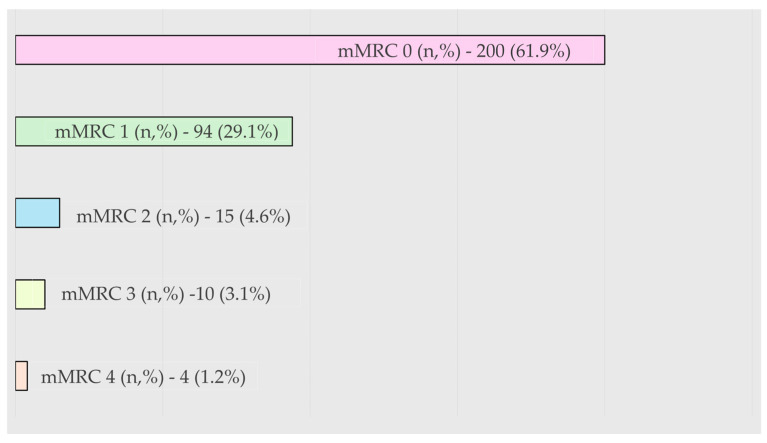
The distribution of mMRC of participants with LDCT-detected emphysema and no lung cancer in the screening cohort.

The median CAT score was nine (range: 0–37), indicating that overall symptoms were mild. Cough, sputum, and exertional breathlessness were the most frequently reported items (Table 2). The distribution of symptoms by the mMRC Dyspnea scale was as follows: mMRC 0: 61.9%; mMRC 1: 29.1%; and mMRC 2–4: 8.9% (Figure 2). Only 7.7% of patients had both mMRC ≥ 2 and CAT ≥ 10, indicating clinically significant symptom burden.

Table 3 summarizes the overlap between airflow limitation, small airways dysfunction, and symptom burden among participants with LDCT-detected emphysema.

### 3.4. Differences in the Presence of Pre-Bronchodilator Airflow Limitation

The differences in lung cancer-screened patients with emphysema according to the presence of (1) pre-bronchodilator airflow limitation, (2) pre-bronchodilator airflow limitation and symptom burden (CAT ≥ 10 and MMRC ≥ 2 vs. CAT < 10 and MMRC 0–1), and (3) pre-bronchodilator airflow limitation and small airways dysfunction (FEF_25–75_ ≥ 60% vs. <60%) are presented in Table 4.

Patients with pre-bronchodilator airflow limitation were more commonly female (*p* = 0.003) and had a lower body mass index (BMI) (*p* = 0.002). They demonstrated significantly lower spirometric parameters, including FEV_1_, FEV_1_/FVC, PEF, and MEF_25/50/75_ (all *p* < 0.001), more frequently met criteria for small airways dysfunction (*p* < 0.001), and exhibited a pathological flow–volume curve (*p* < 0.001).

### 3.5. Pre-Bronchodilator Airflow Limitation with Preserved FEV_1_ (>80%)

Among patients with airflow limitation and preserved FEV_1_ (>80%) (*n* = 101, 70.6%), compared with those without airflow limitation, individuals were more commonly female (57.4%, *p* = 0.020), had a lower BMI (*p* = 0.005), lower FEV_1_, FEV_1_/FVC, PEF, and mid-expiratory flows (all *p* ≤ 0.001), but higher FVC (*p* = 0.008). This group (*n* = 89, 89.9%) also exhibited small airways disease more frequently (*p* < 0.001) and pathological flow–volume curves (*p* < 0.001).

### 3.6. Pre-Bronchodilator Airflow Limitation and Symptom Severity

Patients with more severe symptoms (CAT ≥ 10 and MMRC ≥ 2) were older than those with less severe symptoms (*p* = 0.007), without significant differences in other assessed clinical or functional parameters.

### 3.7. Pre-Bronchodilator Airflow Limitation and Small Airways Dysfunction

Patients with pre-bronchodilator airflow limitation and small airways dysfunction (FEF_25–75_ < 60%), compared with those without small airways dysfunction, were more commonly female (*p* = 0.018) and had significantly lower FVC (*p* = 0.037), FEV_1_, FEV_1_/FVC, PEF, and mid-expiratory flows (all *p* ≤ 0.001), as well as a higher prevalence of pathological flow–volume curves (*p* < 0.001).

### 3.8. Small Airway Dysfunction and Pathological Curves in Patients with Preserved FEV_1_

The differences patients screened for lung cancer with pre-bronchodilator airflow limitation and FEV_1_ > 80% according to the presence of small airways dysfunction (FEF_25–75_ < 60% and FEF_25–75_ ≥ 60%), pathological flow–volume curve, and symptoms (mMRC 0–1 and CAT < 10 and mMRC ≥ and CAT ≥ 10) are presented in Table 5.

**Figure 3:**
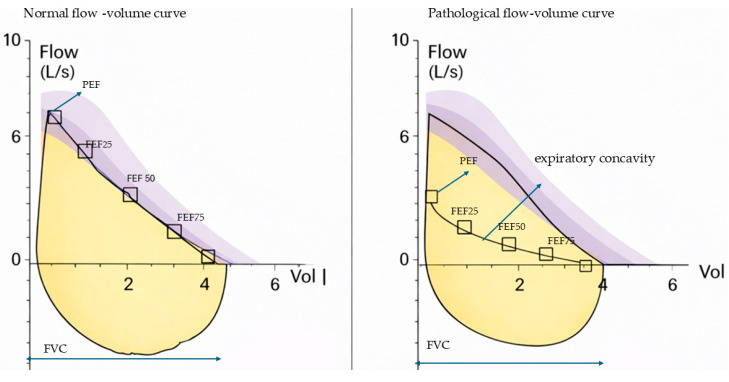
Normal and pathological flow-volume curve.

Patients with small airways dysfunction, in comparison with patients without small airways dysfunction, were more commonly male (*p* = 0.011), had lower spirometric parameters (FEV_1_, FEV_1_/FVC, PEF, MEF_25_, MEF_50_, and MEF_75_) (*p* < 0.001, *p* < 0.001, *p* = 0.031, *p* = 0.001, *p* < 0.001, and *p* < 0.001, respectively), and more frequently had a pathological flow–volume curve (*p* = 0.002). Also, patients with pathological flow–volume curves in comparison with those without had lower spirometric parameters (FVC_1_, FEV_1_, PEF, MEF_25_, MEF_50_, and MEF_75_) (*p* < 0.001, *p* < 0.001, *p* = 0.003, *p* = 0.024, *p* = 0.001, and *p* < 0.001) and more frequently pathological flow–volume curves as well (*p* = 0.002). Finally, patients with more severe symptoms (CAT ≥ 10 and MMRC ≥ 2) were younger than those with less severe symptoms (CAT < 10 and MMRC < 2) (*p* = 0.030). Figure 3 displays the normal and pathological flow–volume curves.

Representative examples of normal and pathological flow–volume curves. The pathological curve demonstrates expiratory concavity (“scooping”), indicative of airflow limitation.

**Figure 4:**
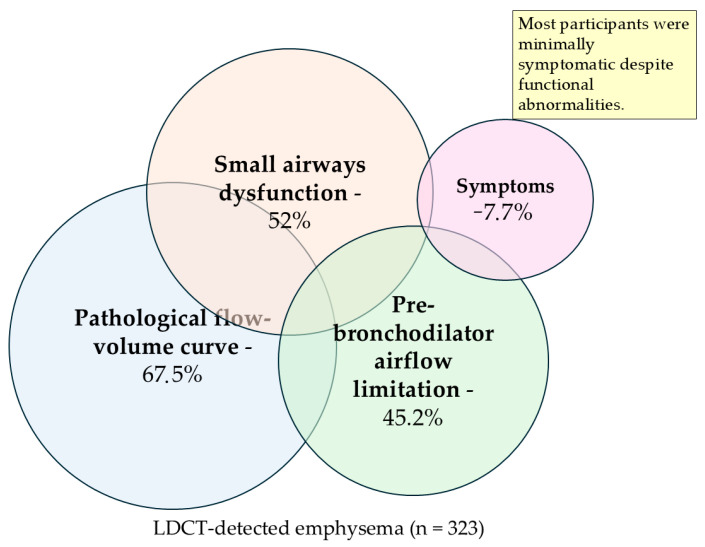
Multidomain functional characterization of participants with LDCT-detected emphysema identified through lung cancer screening.

The overlap between functional impairment and symptom burden in participants with LDCT-detected emphysema is illustrated in Figure 4.

All participants had LDCT-detected emphysema (background). Overlapping circles depict the prevalence of pre-bronchodilator airflow limitation, spirometry-defined small airways dysfunction, pathological flow–volume curve morphology, and clinically significant symptoms. The figure illustrates substantial physiological impairment with only partial overlap between structural, functional, and symptomatic domains. Domains are not mutually exclusive.

## 4. Discussion

This cross-sectional study of participants with LDCT-detected emphysema undergoing spirometry within a lung cancer screening program shows that functional impairment is common, heterogeneous, and frequently present despite preserved FEV_1_ and minimal symptoms. Nearly half of the participants exhibited pre-bronchodilator airflow limitation, while small airways dysfunction and abnormal flow–volume curve patterns were even more prevalent. Importantly, these abnormalities often co-occurred and were observed in minimally symptomatic individuals, including those with preserved FEV_1_.

Although LDCT identifies structural lung abnormalities, it does not capture their physiological consequences. The high prevalence of functional abnormalities in this cohort indicates that spirometry provides complementary information to imaging in screening settings. These findings also suggest that CT-detected emphysema reflects a multidimensional pattern of smoking-related lung abnormalities rather than an isolated structural finding.

Although a substantial proportion of participants demonstrated airflow limitation (FEV_1_/FVC < 0.70), these findings were based on pre-bronchodilator spirometry and should not be interpreted as a diagnosis of COPD but rather as evidence of physiological impairment in a high-risk population. In this context, pre-bronchodilator spirometry should be interpreted as a functional screening tool rather than a diagnostic test. This approach aligns with the objectives of lung cancer screening, where the emphasis is on early risk identification rather than formal disease diagnosis.

To our knowledge, this study is among the first to comprehensively evaluate multiple functional domains—including small airway indices and flow–volume curve morphology—in a screening population with CT-detected emphysema but without a prior respiratory diagnosis.

A key finding of this study is that 52% of participants exhibited small airway dysfunction, including many individuals with preserved FEV_1_. These observations support current ideas that focus on small airways pathology as a key part of early smoking-related lung injury. Hirano et al. (2013) showed that recent evidence indicates that peripheral small airway inflammation and remodeling represent early pathological events in the development of COPD [28]. Verleden et al. (2024) examined lung tissue from patients with emphysematous pre-COPD (*n* = 10), COPD GOLD stages I–IV (*n* = 19 combined), and controls (*n* = 10), finding that small airway numbers were already significantly reduced in pre-COPD patients without airflow obstruction [29]. Chukowry et al. (2021) documented pathological evidence of small airway damage preceding both emphysema development and detection by traditional spirometry [30]. Usmani et al. (2021) demonstrated that significant small airways dysfunction occurs before any overt airway obstruction is detectable by conventional spirometry [31]. Lazarinis et al. (2024) identified small airways as “the primary site for the onset and progression of airflow obstruction” in COPD patients [32].

Small airways are now recognized as the primary site of early inflammatory and structural changes preceding overt airflow obstruction. In this context, the present finding—that individuals with CT-detected emphysema and preserved FEV_1_ frequently demonstrate small airway functional abnormalities—appears highly consistent with this emerging disease model.

Among the functional domains assessed, pathological flow–volume curve abnormalities were the most prevalent, affecting more than two-thirds of the cohort (67.5%). Importantly, abnormal curve morphology was commonly present in individuals without spirometric airflow obstruction, indicating that qualitative assessment of the expiratory curve may capture early functional impairment not reflected by conventional spirometric indices. This finding suggests that flow–volume curve assessment may serve as a sensitive indicator of early airway dysfunction in screening populations. Multiple studies also provide concrete evidence. S. Bhatt et al. (2018) analyzed 8307 COPDGene participants and found that Parameter D (a curve-shaped metric) identified an additional 9.5% of participants as abnormal, with 33.7% of the highest-risk quartile classified as normal by traditional GOLD criteria [33]. D. Johns et al. (2014) noted that extensive small airway disease exists before conventional spirometric detection [34], and three years later, they studied 890 individuals and found that concavity indices approximately doubled the number of subjects detected as abnormal compared to conventional FEF_25–75_ [35].

Symptoms were also generally mild in our population, with only a small proportion reporting clinically significant dyspnea or CAT scores. However, symptom burden correlates with age and, in some cases, with physiological impairment. Obling et al. (2021) also noted that despite high prevalence, small airways dysfunction was not clearly associated with symptom burden in their cohort, suggesting that prevalence alone might not determine clinical symptoms [36]. The predominance of minimally symptomatic individuals underscores the silent nature of early physiological abnormalities. These findings also highlight the limitations of symptom-based approaches in detecting early disease in screening populations. These findings support the potential role of lung cancer screening programs complemented by spirometry as a practical setting for identifying asymptomatic but physiologically vulnerable smokers.

Sex-related differences also emerged. Women were more likely to demonstrate pre-bronchodilator airflow limitation, whereas men more frequently exhibited small airway dysfunction. These findings raise the possibility of sex-specific characteristics in early functional impairment, consistent with recent literature describing gender differences in susceptibility to smoking-related lung injury. Sørheim et al. (2010) demonstrated that female gender is associated with reduced lung function and more severe disease in subjects with COPD who have early disease onset or low smoking exposure [37]. A. Steinberg et al. (2025) [38] showed that women aged 40 and above have about a 50% higher risk of COPD compared to men. Still, this difference cannot be explained by women’s greater susceptibility to cigarette smoking. Lower BMI among patients with pre-bronchodilator airflow limitation further suggests early systemic involvement or differences in body composition that may influence lung mechanics. Multiple studies demonstrate the relationship between BMI and respiratory disease. Trethewey et al. (2022) found lower BMI trajectories throughout adulthood among patients with airflow obstruction and emphysema, with 4587 participants, suggesting that body mass may precede COPD development [39].

The study has several limitations. Its cross-sectional design precludes assessment of longitudinal outcomes and progression to clinically overt disease. Spirometry was performed without post-bronchodilator testing; therefore, persistent airflow limitation could not be confirmed, and findings should be interpreted as physiological impairment rather than a diagnosis of COPD. The assessment of small airways dysfunction was based on mid-expiratory flow parameters, which are known to exhibit variability and dependence on lung volumes, particularly FVC, potentially limiting their specificity when used in isolation. In addition, flow–volume curve morphology was evaluated qualitatively, and interobserver variability was not formally assessed. Finally, radiological assessment of emphysema was based on a dichotomous classification using a standard densitometric threshold, without detailed quantification of emphysema extent. No selection bias occurred at the level of spirometry participation; however, potential selection bias related to initial screening enrollment cannot be excluded.

Taken together, these findings describe the presence of physiological abnormalities in individuals with CT-detected emphysema identified through lung cancer screening, including those with preserved FEV_1_ and minimal symptoms. These findings may have implications for the integration of functional assessment into lung cancer screening pathways. Longitudinal follow-up of this cohort will be essential to determine the clinical relevance of these findings and their potential association with future respiratory outcomes.

## 5. Conclusions

In a lung cancer screening (LCS) cohort with CT-detected emphysema, multidimensional functional impairment is common and frequently present despite preserved FEV_1_ and minimal symptoms. Pre-bronchodilator airflow limitation, small airways dysfunction, and abnormal flow–volume curve morphology were frequently observed across the cohort, including in individuals without significant symptoms. Spirometry provides additional physiological information beyond structural imaging; however, these findings should be interpreted as descriptive and not diagnostic of COPD. Further longitudinal and analytical studies are warranted to determine the clinical relevance of these findings and their potential association with future respiratory outcomes.

## Figures and Tables

**Table 1:** Pre-bronchodilator spirometry characteristics of participants with LDCT-detected emphysema and no lung cancer in the screening cohort.

Spirometry Parameter	Mean ± SD (% of Predicted)
FVC (%predicted)	114.5 ± 17.2
FEV_1_ (%predicted)	98.9 ± 18.7
FEV1/FVC (%predicted)	69.6 ± 8.5
PEF (%predicted)	97.7 ± 20.6
	Median (min–max) (% of predicted)
MEF_25_ (%predicted)	38 (9–129)
MEF_50_ (%predicted)	57 (12–167)
MEF_75_ (%predicted)	88 (18–155)
FEF_25–75_ (%predicted)	
≥60%	155 (48.0)
<60%	168 (52.0)
Flow-volume curve	*n* (%)
Normal	105 (32.5)
Pathological	218 (67.5)

Abbreviations: FVC, forced vital capacity; FEV_1_, forced expiratory volume in one second; PEF, peak expiratory flow; MEF_25/50/75_, maximal expiratory flow at 25%, 50%, and 75% of FVC; FEF_25–75_—forced expiratory flow between 25% and 75% of FVC.

**Table 2:** COPD Assessment Test (CAT) scores in participants with CT-detected emphysema in the lung cancer screening cohort.

CAT Questions	Median (Min–Max)
Cough	2 (0–5)
Phlegm/mucus	2 (0–5)
Chest tightness	0 (0–5)
Breathlessness (hills/stairs)	2 (0–5)
Activities at home	0 (0–5)
Confidence leaving home	0 (0–5)
Sleep	1 (0–5)
Energy	1 (0–5)
Total score	9 (0–37)

**Table 3:** Overlapping functional and symptomatic characteristics among participants with LDCT-detected emphysema and no lung cancer.

Overlapping Functional and Symptomatic Characteristics	*n* (%)
Emphysema + airflow limitation	146 (45.2)
Emphysema + airflow limitation + pathological curve	127 (39.3)
Emphysema + airflow limitation (FEV_1_ ≥ 80%)	105 (32.5)
Emphysema + no airflow limitation + pathological curve	90 (27.9)
Emphysema + no airflow limitation + normal curve	87 (26.9)
Emphysema + mild symptoms + airflow limitation + pathological curve	68 (21.1)
Emphysema + mild symptoms + airflow limitation + pathological curve + small airways dysfunction	68 (21.1)
Emphysema + significant symptoms	25 (7.7)
Emphysema + no airflow limitation + mild symptoms + small airways dysfunction	19 (5.9)
Emphysema + significant symptoms + airflow limitation + pathological curve	5 (1.5)
Emphysema + significant symptoms + airflow limitation + pathological curve + small airways dysfunction	5 (1.5)
Emphysema + no airflow limitation + significant symptoms + small airways dysfunction	0 (0.0)

Abbreviations: airflow limitation refers to pre-bronchodilator airflow limitation (FEV_1_/FVC < 0.70); small airways dysfunction is defined as FEF_25–75_ < 60%; mild symptoms are defined as mMRC 0–1 and CAT < 10; significant symptoms are defined as mMRC ≥ 2 and CAT ≥ 10. Categories are not mutually exclusive.

**Table 4:** Subjects with LDCT evidence of emphysema and no lung cancer in the LCS program, according to the pre-bronchodilator airflow limitation, the severity of symptoms, and the presence of small airways dysfunction.

Characteristic	Airflow Limitation		*p*	Airflow Limitation (FEV1 ≥ 80%)		*p*	Small Airway Dysfunction		*p*
	Yes *n* = 146	No *n* = 177		No *n* = 177	Yes *n* = 101		Yes *n* = 134	No *n* = 12	
Baseline									
Age, years	65.7 ± 6.9	64.8 ± 8.1	0.309	64.8 ± 8.1	65.4 ± 6.9	0.527	65.5 ± 6.8	68.2 ± 7.0	0.194
Male	59 (40.4)	101 (57.1)	**0.003**	101 (57.1)	43 (42.6)	**0.020**	76 (56.7)	11 (91.7)	**0.018**
Female	87 (59.6)	76 (42.9)		76 (42.9)	58 (57.4)		58 (43.3)	1 (8.3)	
BMI, kg/m^2^	25.3 ± 4.6	26.9 ± 4.2	**0.002**	26.9 ± 4.2	25.4 ± 4.0	**0.005**	25.2 ± 2.7	27.1 ± 3.9	0.165
Spirometry									
FVC	113.5 ± 17.1	115.3 ± 17.3	0.365	115.3 ± 17.3	120.6 ± 13.4	**0.008**	112.7 ± 17.1	123.3 ± 13.4	**0.037**
FEV_1_	88.3 ± 16.1	107.2 ± 16.2	**<0.001**	107.6 ± 16.2	96.7 ± 10.8	**<0.001**	86.5 ±15.3	108.7 ± 10.4	**<0.001**
FEV_1_/FVC	62.1 ± 6.2	75.7 ± 3.8	**<0.001**	75.7 ± 3.8	64.3 ± 4.3	**<0.001**	61.6 ±6.2	68.3 ± 1.3	**<0.001**
PEF	89.3 ± 21.6	104.5 ±17.0	**<0.001**	104.5 ± 17.0	97.4 ± 19.2	**0.001**	87.6 ± 21.4	108.5 ± 13.4	**0.001**
MEF_25_	27 (9–65)	49 (17–129)	**<0.001**	49 (17–129)	31 (15–65)	**<0.001**	27 (9–54)	38.5 (27–65)	**<0.001**
MEF_50_	38.5 (12–73)	77 (32–167)	**<0.001**	77 (32–167)	44 (24–73)	**<0.001**	37 (12–59)	64.5 (53–73)	**<0.001**
MEF_75_	68 (18–127)	99 (29–155)	**<0.001**	99 (29–155)	78 (33–127)	**<0.001**	63.5 (18–123)	99.5 (68–127)	**<0.001**
FEF_25–75_									
<60%	134 (91.8)	34 (19.2)	**<0.001**	34 (19.2)	89 (88.1)	**<0.001**	NA	NA	NA
≥60%	12 (8.2)	143 (80.8)		143 (80.8)	12 (11.9)		NA	NA	
Flow- volume curve, *n* (%)									
Normal	18 (12.3)	87 (49.2)	**<0.001**	87 (49.2)	18 (17.8)	**<0.001**	12 (9.0)	6 (50.0)	**<0.001**
Pathological	128 (87.7)	90 (50.8)		90 (50.8)	83 (82.2)		122 (91.0)	6 (50.0)	

*p*-values were calculated using Student’s *t*-test, Mann–Whitney U test, chi-square test, and Fisher’s exact test, where appropriate. Values are presented as mean ± standard deviation (SD) or median (minimum–maximum), as appropriate. Airflow limitation refers to pre-bronchodilator airflow limitation (FEV_1_/FVC < 0.70). Small airways dysfunction is defined as FEF_25–75_ < 60%. Spirometry variables are expressed as a percentage of predicted values (% predicted). Abbreviations: FVC, forced vital capacity; FEV_1_, forced expiratory volume in one second; PEF, peak expiratory flow; MEF_25/50/75_, maximal expiratory flow at 25%, 50%, and 75% of FVC; FEF_25–75_—forced expiratory flow between 25% and 75% of FVC; NA, not applicable. Bold values indicate statistically significant differences (*p* < 0.05).

**Table 5:** Participants with emphysema, airflow limitation with preserved FEV_1_ (≥80%), according to the presence of small airways dysfunction and pathological flow–volume curve morphology.

Characteristic	Small Airways Dysfunction		*p*	Flow–Volume Curve		*p*
	Yes *n* = 89	No *n* = 12		Normal *n* = 18	Pathological *n* = 83	
Baseline						
Age, years	65.1 ± 6.9	68.2 ± 7.0	0.146	68.1 ± 6.1	64.9 ± 7.0	0.076
Gender						
Male	42 (47.2)	1 (8.3)	**0.011**	7 (38.9)	36 (43.4)	0.727
Female	47 (52.8)	11 (91.7)		11 (61.1)	47 (56.6)	
BMI, kg/m^2^	25.2 ± 4.0	27.1 ± 3.9	0.123	24.6 ± 3.6	25.6 ± 4.1	0.363
Spirometry						
FVC	120.2 ± 13.4	123.3 ± 13.4	0.454	138.8 ± 13.3	116.7 ± 9.6	**<0.001**
FEV_1_	95.1 ± 9.8	108.7 ± 10.4	**<0.001**	113.3 ± 6.8	93.1 ± 7.6	**<0.001**
FEV_1_/FVC	63.8 ± 4.3	68.3 ± 1.3	**<0.001**	65.2 ± 4.2	64.2 ± 4.3	0.376
PEF	95.9 ± 19.4	108.5 ± 13.4	**0.031**	109.3 ± 27.5	94.8 ± 15.9	**0.003**
MEF_25_	30 (15–54)	38.5 (27–65)	**0.001**	35 (23–65)	30 (15–54)	**0.024**
MEF_50_	42 (24–59)	64.5 (53–73)	**<0.001**	55 (37–73)	42 (24–68)	**0.001**
MEF_75_	76 (33–123)	99.5 (68–127)	**<0.001**	91.5 (77–123)	74 (33–127)	**<0.001**
FEF_25–75_						
<60%	NA	NA	NA	12 (66.7)	77 (92.8)	**0.002**
≥60%	NA	NA		6 (33.3)	6 (7.2)	
Flow-volume curve, *n* (%)						
Normal	12 (13.5)	6 (50.0)	**0.002**	NA	NA	NA
Pathological	77 (86.5)	6 (50.0)		NA	NA	

*p*-values were calculated using Student’s *t*-test, Mann–Whitney U test, Chi-square test, and Fisher’s exact test, as appropriate. Values are presented as mean ± standard deviation (SD) or median (minimum–maximum), as appropriate. Airflow limitation refers to pre-bronchodilator airflow limitation (FEV_1_/FVC < 0.70). Small airways dysfunction is defined as FEF_25–75_ < 60%. Spirometry variables are expressed as a percentage of predicted values (% predicted). Abbreviations: BMI—Body Mass Index; FVC, forced vital capacity; FEV_1_, forced expiratory volume in one second; PEF, peak expiratory flow; MEF_25/50/75_, maximal expiratory flow at 25%, 50%, and 75% of FVC; FEF_25–75_—forced expiratory flow between 25% and 75% of FVC; NA, not applicable. Bold values indicate statistically significant differences *(p* < 0.05).

## Data Availability

The data supporting the findings of this study are not publicly available due to patient confidentiality and ethical restrictions but are available from the corresponding author upon reasonable request and with appropriate ethical approval.

## Abbreviations

LDCT	Low-Dose Computed Tomography
LCS	Lung Cancer Screening
BMI	Body Mass Index
FVC	Forced Vital Capacity
FEV_1_	Forced Expiratory Volume in One Second
FEV_1_/FVC	Ratio of Forced Expiratory Volume in 1 s to Forced Vital Capacity
PEF	Peak Expiratory Flow
MEF_25_	Maximal Expiratory Flow at 25% of FVC
MEF_50_	Maximal Expiratory Flow at 50% of FVC
MEF_75_	Maximal Expiratory Flow at 75% of FVC
FEF_25–75_	Forced Expiratory Flow Between 25% and 75% of FVC
COPD	Chronic Obstructive Pulmonary Disease
CAT	COPD Assessment Test
mMRC	Modified Medical Research Council Dyspnea Scale
